# Cytotoxicity evaluation, porosity and physicochemical study of bioactive glass nanocomposites for tissue engineering

**DOI:** 10.1016/j.toxrep.2026.102329

**Published:** 2026-08-20

**Authors:** Mohammad Shokrzadeh, Nazanin Farhadyar, Elahe Gharekhani, Fereshteh Fathi, Masoumeh Eslamifar

**Affiliations:** a Department of Toxicology-Pharmacology, Faculty of Pharmacy, Mazandaran University of Medical Sciences, Sari, Iran; b Department of Chemistry, Islamic Azad University, Varamin-pishva Branch, Varamin, Iran; c Department of environmental health engineering, Faculty of Health, Mazandaran University of Medical Sciences, Sari, Iran

**Keywords:** Alginate, Nano bioactive glass, MTT assay, Composite, Tissue engineering

## Abstract

Nanoscale bioactive glasses have been developed as regenerative materials for tissue engineering, due to their biocompatibility, osteoconductivity, antibacterial properties and abilities for hard tissue formation. In this work, nano bioactive glass/alginate (n-BG/Alg) and nano bioactive glass/polyethylene glycole (n-BG/PEG) as nanocomposite scaffolds were synthesized via facile method and physicochemical properties compared with nano bioactive glass. To characterize the nanocomposites, Fourier Transform Infrared (FT-IR), X-ray powder Diffraction (XRD), Field Emission Scanning Electron Microscopy (FESEM), and Dispersive X-ray Analysis (EDX) were performed. The results showed desired interaction of polymers with nano bioactive glass and the size was estimated. The swelling ability and porosity of the scaffolds were evaluated and the results showed lower swelling and porosity percentage for composites compared to the nano bioactive glass scaffold. The cytocompatibility of nanocomposites were also evaluated using Methyl Thiazolyl-Tetrazolium (MTT) (3-(4, 5-dimethyl-2-thiazolyl)-2, 5-diphenyl-2H-tetrazolium bromide) assay. The doses of 5, 15, 25, 50, and 100 μg/mL of n-BG, n-BG/PEG and n-BG/Alg were studied to determine the survival rate of Human Gingival Fibroblast (HGF) cells. The results showed a significant difference (p˂0.001) compared to the positive control group in high concentrations during 48 h. The MTT assay confirms that the scaffolds have biocompatibility.

## Highlights

• Nano bioactive glass/alginate (n-BG/Alg) and Nano bioactive glass/PEG (n-BG/PEG) composites scaffolds synthesized via sol-gel method.
• Fourier Transform Infrared (FT-IR), X-ray Diffraction (XRD), Field Emission Scanning Electron Microscopy (FESEM), Dispersive X-ray Analysis (EDX) confirmed the size and morphology of the nanocomposite scaffolds. Porosity and water uptake ability was determined by liquid displacement method employing Archimedes’ Principle.
• Cytotoxicity of the nanostructures was evaluated using Human Gingival Fibroblast (HGF) cell model, by Methyl Thiazolyl-Tetrazolium (3-(4, 5-dimethyl-2-thiazolyl)-2, 5-diphenyl-2H-tetrazolium bromide) (MTT) assay.
• These studies indicated biocompatibility of the n-BG/PEG composite scaffold on HGF cell line.

## Graphical Abstract

**Figure:**
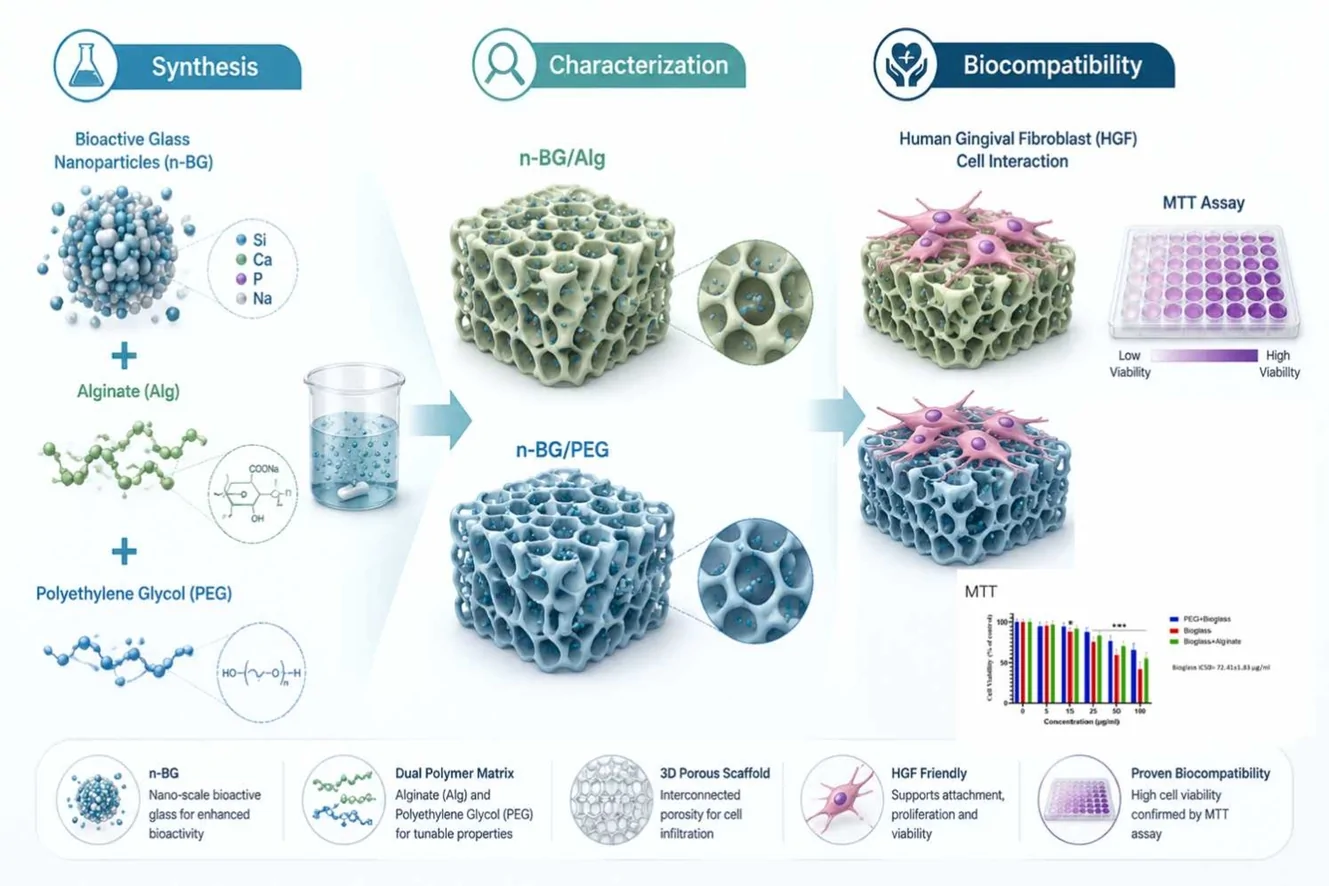

## 1 Introduction

Dental tissue engineering is a novel technique for regeneration and replacement of damaged or missing tooth. Advances in this field have been reported to aim the regeneration of dental tissues via biocompatible scaffolds as matrix [1]. Biomaterial scaffolds are temporary synthetic three-dimensional substrates that designed to mimic the extra cellular matrix (ECM) and support cell attachment, growth, and differentiation [2]. Polymer composite scaffolds were proposed as extracellular matrix supports for bone, cartilage, and ligament regeneration [3].

Nanocomposite scaffolds developed from natural and synthetic polymers are commonly used in dental tissue engineering. Bioactive glass (BG)-based polymer nanocomposites as 3D scaffolds exhibit strong interactions with hard tissues, forming stable interfaces after implantation [4], [5].

Nano bioactive glass (n-BG) has shown promising results in dental applications, particularly for remineralization and caries prevention. Studies have demonstrated that n-BG composites improve the alkalizing potential of dental composites without negatively impacting fundamental properties like degree of conversion and micro hardness [6], [7]. n-BG exhibit faster formation of calcium phosphate layers, enhancing their bioactivity [8]. When combined with biocompatible polymers, n-BG create nanocomposites with improved physicochemical, mechanical, biological properties of scaffolds and osteoblast cell attachment for orthopedic applications [9]. The shape and size of pores, porosity, and interconnectivity of scaffolds are critical determinants of tissue formation. In remineralization studies, n-BG incorporated in to adhesives has shown higher remineralizing potential than nanohydroxyapatite and sodium fluoride, particularly under neutral conditions [10].

Alginate-based composites have been developed to overcome limitations of native alginate, improving mechanical properties and promoting tissue regeneration [11] alginate’s ability to form hydrogels makes it suitable for 3D printing applications in medical, with various composite combinations enhancing its properties [12], [13] Recent research has explored the use of polymer-based nanocomposites for dental applications [14], [15], [16], [17].The researches have shown that this scaffold exhibit appropriate pore sizes and swelling behavior for cell adhesion, proliferation and apatite formation. These supporting effects can be control with the BG content [18].

Polyethylene glycol (C_2n_H_4n+2_O_n+1,_ PEG) is a synthetic, soluble, and environmentally friendly polymer that interact with biological systems [19]. PEG is valuable in various applications, including drug delivery and tissue engineering, due to its non-toxicity, cytocompatibility, osteoconductivity, protein interactions and biodegradability [20], [21], [22].

PEG is commonly incorporated into bioactive glasses, affect its physical and biological properties, including pore size, crystallinity, surface structure, degradation rate, and cell proliferation [23], [24]. The molecular weight and concentration of PEG significantly influence the morphology and size of BG nanoparticles, with higher molecular weight PEG producing smaller particles [25]. The n-BG/polymer composites can be fabricated using various methods, including hydrothermal, sonochemical, sol-gel processes, electrospinning, and freeze-drying techniques [26], [27].

Lee et al. investigated sol-gel synthesis of mesoporous bioactive glass nanoparticles and amination by APTES for efficiency of nanoparticles with their endocytosis pathway on rat dental pulp stem cells via invert negative surface charge to positive [4]. The ultrasound irradiated bioglass synthesis was performed within a shorter time period compared with conventional synthesis. Ultrasonic irradiation was performed in an (Ultrasonics) ultrasonic processor, with a power of 180 W and a frequency of 24 kHz, under 70% amplitude and power rotary cycles of 1.0 s. Recent researches have focused on synthesizing BG nanocomposites with polymers, such as PEG, alginate, and PVP for biomedical applications. These nanocomposites exhibit enhanced bone cell proliferation, ALP activity, and apatite mineralization compared to polymer-only hydrogels [28], [29]. The combination of n-BG with biodegradable polymers offers improved physicochemical, mechanical, and biological properties, making them promising materials for orthopedic applications and tissue engineering scaffolds [29].

It is notable that the particle size and surface morphology of the bioactive glass nanoparticles (n-BG) play an important role for biological purposes. Chen et al. [30] reported that the using of lactic acid (LA) in the CaO-P2O5-SiO2 bioactive glass system cause to decrease the particles size of the bioactive glass nanoparticles and improve the surface morphologies for in vitro applications. Kazemi et al., 2023 investigated surface modification of silica nanoparticles with polyethylene glycol, PEG, in acrylic dental composites has demonstrated protein-repellent and antibacterial properties [31]. In another study, Taymour et al., 2022 showed the incorporation of nanofillers has generally led to increased surface roughness and flexural strength in dental composites [32]. These nanocomposites have demonstrated improved initial cell adhesion, proliferation, and mineralization compared to unmodified materials. These advancements suggest that polymer-based nanocomposites have significant potential for various dental applications, including implants and restorative materials. Table 1 listed some of these n-BG/polymer composites via various methods for tissue engineering.

**Table 1:** n- BG composites for tissue engineering.

Composition	Fabrication	Advantages
n-BG/PEEK* [32], [33]	Melt blending, molding	Osseointegration and mechanical strength
n-BG [34]	Sonochemical, hydrothermal and sol–gel	Higher bone-bonding ability via sol–gel method
n-BG/APTES^**^[35]	Sol-gel	promoting degradation,cell viability, proliferation, and attachment
n-BG/fibrin [36]	Sol-gel	Stability, Reduces the degradation rate, Improves cell viability, cell proliferation
n-BG/Alginate/Xanthan [37]	Sol-gel	aggregation, reduced antioxidant properties, Increased hemolysis
n-BG/Alginate/Gelatin [38]	Freeze-drying	Biocompatibility, High porosity, Good swelling capacity, and biodegradability, Improved the osteoblastic function and bone volume fraction
n-BG/Strontium/Zinc [39]	Microemulsion-assisted Sol–gel	Induce osteogenic, Antibacterial properties, promoting bone regeneration
n-BG/P3HB^***^[40]	Sol-gel and Electrospinning	High porosity, Distribution
n-BG/gelatin-collagen [36]	Sol-gel	Differentiation of EnSCs, Increased VEGF expression
n-BG/fibroin/carboxymethylcellulose[41]	Electrospinning	Biodegradability, Bioactivity, Improved adhesion, proliferation and viability of cells, osteogenic differentiation biomineralization,

* Polyether-ether-ketone, ** PLA (3-Aminopropyl) triethoxysilane, *** Poly (3-hydroxybutyrate).

## 2 Materials and methods

Calcium nitrate [Ca (NO_3_)_2_.4H_2_O], Ammonium hydrogen phosphate [(NH_4_)_2_HPO_4_], Tetraethoxysilane [TEOS], Nitric acid [HNO_3_], and Ethanol [CH_3_CH_2_OH] purchased from Merck, Germany. Poly Ethylene Glycol (PEG 4000) and Sodium alginate purchased Sigma-Aldrich. Table 2 summerize the origin and purity of these precursors. All solvents were used without more purification. Distilled water used in all experimental process. Also, Dulbecco’s Modified Eagle’s Medium (DMEM), and 3-(4, 5-Dimethylthiazol−2-yl)−2, 5-diphenyl-tetrazoliumbromide) (MTT) were purchased from Sigma–Aldrich (USA). The Human Gingival Fibroblast (HGF) was purchased from the Pasteur Institute of Iran. 10% Fetal Bovine Serum (FBS, Thermo Scientific, USA), and Penicillin and Streptomycin (Thermo Fischer Scientific, Waltham, MA, USA) were used for In- Vitro MTT assay. Table 2 listed the origin and purity of these precursors.

**Table 2:** The origin and purity of precursors.

Chemical name	Source	Mole fraction purity
Tetraethyl orthosilicate*	Merck	0.98
Calcium nitrate tetrahydrate **	Merck	0.99
Ethanol	Merck	0.99
Nitric acid (HNO_3_)	Merck	˃ 0.98
Ammonium dihydrogen phosphate***	Merck	0.98
Polyethylenglycol	Sigma-aldrich	0.98
Sodium alginate	Sigma aldrich	0.98

*:(TEOS, SiC_8_H_20_O_4_), **: (Ca(NO_3_)_2_.4H_2_O), ***: (NH_4_(H_2_PO_4_).

### 2.1 Nano bioactive glass (n-BG) synthesis

**Step1- Sol formation:**

In the clean beaker, 20 mL of ethanol and 1–2 mL of dilute nitric acid (2 N) mixed. TEOS slowly added to this solution. The solution stirred magnetically for 30 min (room temperature, 300 rpm). 1% (W/V) of PEG−4000 added to the solution and continued stirring until clear-PEG controls porosity and flexibility.

**Step 2- Adding Calcium and Phosphorus precursors:**

Dissolve Ca (NO₃)₂•4H₂O and (NH₄)₂HPO₄ in 10 mL of water separately and add dropwise to the TEOS–PEG solution. Stir continuously until the mixture is completely homogeneous (about 1–2 h).

**Step 3- Gelation and aging:**

Pour the solution into a covered container and keep at room temperature for 24 h to form a gel. Then age it at 60°C for 24 h to remove the solvents.

**Step 4 - Drying and heating:**

Place the dried gel in a 100°C drying oven for 12 h. Then grind the powder and slowly calcined at 600°C for 3 h to form a silicate network (in the presence of PEG, a porous and relatively elastic structure is obtained). The synthesized composite material was further used for gel-casting to prepare scaffold structure for bone tissue engineering applications.

Ref

Preparation and characterization of nano-bioactive-glasses (NBG) by a quick alkali-mediated sol–gel method

Synthesis, characterization and biological response of magnesium-

substituted nanobioactive glass particles for biomedical applications

Advantages of nanoscale bioactive glass as inorganic filler in alginate hydrogels for drug delivery and biofabrication

Preparation and in vitro evaluation of polycaprolactone/PEG/bioactive glass nanopowders nanocomposite membranes for GTR/GBR applications

### 2.2 Characterizations

#### 2.2.1 FT-IR

Fourier transformed Infra-Red spectroscopy (FT-IR) has been used to study of structure and functional groupings of nanobioactive glass (n-BG), nanobioactive glass/alginate (n-BG/Alg) and nanobioactiveglass/polyvinylpyrrolidone (n-BG/PVP) nanocomposites. To determine potential formation of new functional groups in these nanocomposites, samples were embedded in KBr pellets and recorded by FT-IR (Thermo Fisher SCIENTIFIC, Nicolet Is5) spectrometer between 4000 cm^−1^ - 400 cm^−1^ in transmittance mode with a resolution of 2 cm^−1^.

#### 2.2.2 XRD

X-Ray Diffraction (XRD, PANalytical Empyrean) was used to confirm nanostructure formation. XRD analyses were carried out at ambient temperature, scan specimens in the range of two-theta (2θ) diffraction angles, from 10 to 80 °, at a rate of 2 °/min, with a step-width of 0.02 ° using Cu Kα1 radiation at 40 kV and a current strength of 40 mA. The crystalline size of the n-BG and nanocomposites was calculated by the Scherer equation as follows:

(1) $$D=\frac{K\lambda }{\beta 1/2*\cos\theta }$$

Where D is the average crystal size, K is a constant (here chosen as 1), λ is the wavelength of X-ray radiation (1.54056 A), β_1/2_ is the half width of the diffraction peak, and θ (◦) is Bragg’s angle.

#### 2.2.3 FESEM & EDX

Morphological studies of nanostructures (n-BG and nanocomposites) are done by Field Emission Scanning Electron Microscope (FESEM, Nova NanoSEM 450) operated at an accelerating voltage of 10 kV. Energy Dispersive X-ray spectroscopy nanocomposites (EDX, Rantek, America) is utilized to determine the elemental composition (purity) of prepared nanocomposites. As a result, the obtained nanocomposite structure is very important for medical applications.

#### 2.2.4 Porosity and water uptake study

The porosity A (%) of n-BG and nanocomposites was determined according to our previous studies by liquid displacement method [42], [43], [44]. For this purpose, at first, the n-BG and nanocomposite scaffolds were separately immersed in ethanol with initial volume (v1) and vacuum situation with no air penetration for 48 h until they get fully saturated. Their saturated volume was recorded (V2). The ethanol was then removed and the remaining volume of ethanol was defined as (V3). The volume of the scaffolds (V2 − V1). The porosity A (%) of each scaffold was then calculated using the formula bellow:

(2) $$A\left(\%\right)=\frac{V1-V3}{V2-V3}\times 100$$

For water uptake assay, the dry weight of scaffolds was estimated separately and recorded as (W1). Then, the scaffolds immersed in distilled water at room temperature for 48 h. the scaffolds were then removed and the wet weight was recorded as (W2). The capacity of water absorption (W.A) of the scaffolds was determined using Eq. (3):

(3) $$W.A\left(\%\right)=\frac{W2-W1}{W1}\times 100$$

#### 2.2.5 MTT assay

The *in vitro* biocompatibility of the n-BG, n-BG/Alg and n-BG/PEG composites was evaluated on Human Gingival Fibroblast (HGF) cell model, using yellow and water soluble Methyl Thiazolyl-Tetrazolium salt (MTT) (3-(4, 5-dimethyl−2-thiazolyl)−2, 5-diphenyl-2H-tetrazolium bromide) assay. As our previous work, cell suspensions containing Dulbecco's modified Eagle's medium (DMEM), 1% pen-strep, and 10% fetal bovine serum (FBS) were placed in a 75-cm3 cell culture flask and incubated at 37°C in a humid atmosphere of 5% CO2 to reach their logarithmic growth stage. When the primary culture became almost confluent, 1 mL of trypsin-EDTA was added to the flask and incubated for 5 min to completely separate the cells from the flask. Then, to evaluate the oxidative stress and cell survival tests, 100 μl of cell suspension was added to each well in 96-well plates, and the plates were incubated for 24 h. After 24 h of incubation, the cell line was exposed to doses of 5, 15, 25, 50 and 100 μg/mL of n-BG, n-BG/Alg and n-BG/PEG scaffolds with incubation for 48 h. Then, 10 μl of MTT solution was added to each well and incubated for 4 h. After that the contents of each well were removed and washed with phosphate-buffered saline, then 30 μl of dimethyl sulfoxide (DMSO) was added to dissolve the formazan dye, and absorption was determined at max = 490 and 630 nm using the BioTek ELx800 microplate reader. The Graph Pad Prism (Version 3) was used to calculate data (mentioned in results) [44], [45], [46].

## 3 Results and discussions

For dental tissue engineering, scaffolds with appropriate strength, rigidity, sufficient cell growth space, and permeability is required. To date, nanobioactive glasses with polymer matrixes have been widely used for cell culture scaffolds. In this study, n-BG and n-BG/PEG and n-BG/Alg composite scaffolds were synthesized and investigated for tissue engineering purposes. Some physicochemical properties are investigated with characterization techniques.

### 3.1 FT-IR

**Fig. 1:**
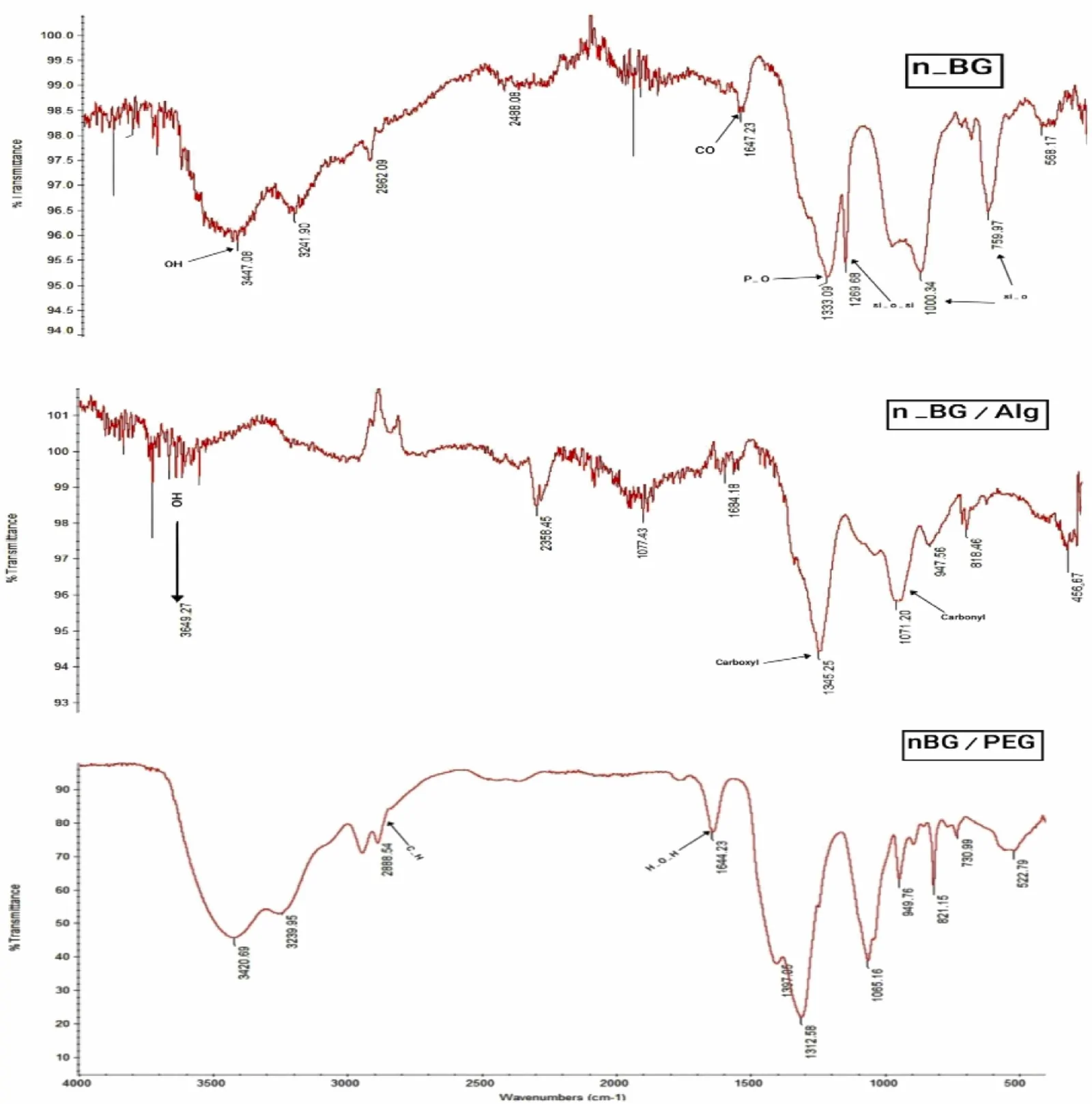
FT-IR of n-BG, n-BG/Alg and n-BG/PEG composite scaffolds.

The FTIR spectra of n-BG, n-BG/PEG, and n-BG/Alg composites were shown in Fig. 1. The peak at 3447.08 cm^−1^ is due to OH stretching vibration. The peaks at 1647.23 and 1333.09 cm^−1^ indicated the existence of C-O and P-O groups in the structure. The symmetric stretching vibration bands of Si-O were observed at 1000.34 and 759.97 cm^−1^[47]. Also, the band at 568.17 cm^−1^ and a shoulder at 1269.68 cm^−1^ indicated Si–O–Si bending mode [30], [48], [49]. The peaks at 2888.54 and 1644.23 cm^−1^ are attributed to C-H stretching and H-O–H (molecular water) bending vibration band of PEG. This indicates the interaction of PEG with n-BG.

FT-IR spectra of n-BG/Alg nanocomposite scaffold showed the combined peaks of alginate and n-BG which confirmed the chemical interactions between bioactive glass and alginate. As seen, FT-IR spectra of alginate scaffold showed a peak around 3420 cm^−1^ indicates the absorption of O–H group. A peak around 1345.25 cm^−1^ is due to the presence of carboxyl group. Peaks at 1071.20 cm^−1^ indicate to the presence of carbonyl groups. Table 3 listed FT-IR main bands of scaffolds ().

**Table 3:** Summarized the some characterization bands in FT-IR spectra.

Nanostructure	Band	Characterization
n-BG	3447.08 cm^−1^	O-H Stretching
	1647.23 cm^−1^	C-H Stretching
	1333.09 cm^−1^	P-O Stretching
	1000.34, 759.97 cm^−1^	Si-O Stretching
	1269.68, 568.17 cm^−1^	Si-O-Si Bending
n-BG/Alg	2888.54 cm^−1^	C-H Stretching
	1644.23 cm^−1^	H-O-H Bending
n-BG/PEG	1345.25 cm^−1^	COO^-^ Group
	1071.20 cm^−1^	C=O^-^ Group

**Table 4:** Quantitative Results of n-BG, n-BG/Alg and n-BG/PEG.

	n-BG %	n-BG/Alg %	n-BG/PEG %
**N**	5.23	31.8	15.6
**Si**	80.2	19.5	18.0
**P**	14.4	17.5	24.6
**C**	0	0	41.7
**Na**	0	31	0

### 3.2 XRD

**Fig. 2:**
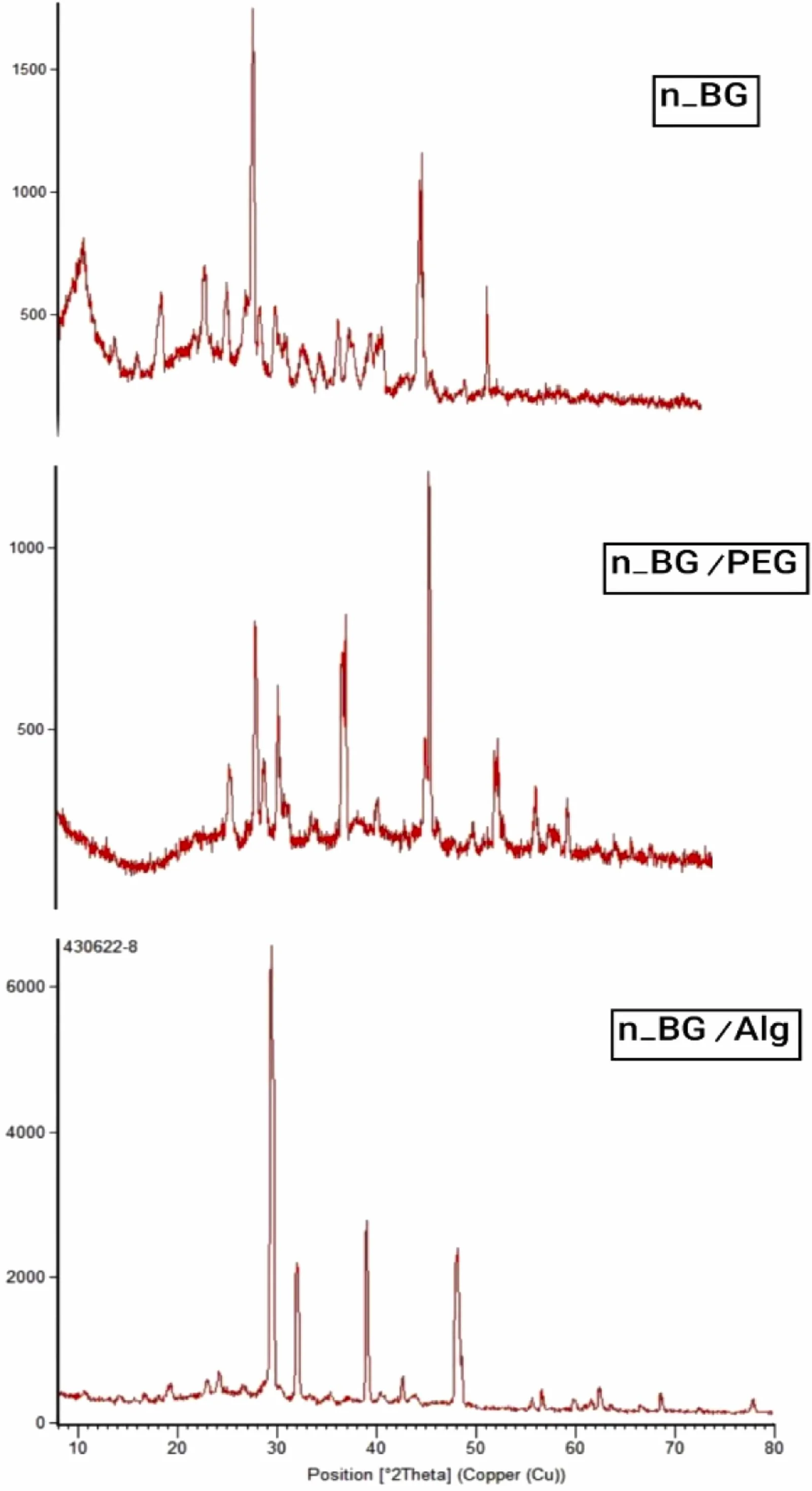
XRD of the n-BG, n-BG/Alg and n-BG/PEG composite scaffolds.

The crystalline structural analysis of nano bioactive glass and nanocomposites showed in Fig. 2. The XRD spectra of n-BG confirmed that they generally exist in highly crystalline state.

The 2*θ* scanning range was set between 8° and 80°. It is noteworthy that the diffraction peaks appeared around 2*θ* = 29.76°, 48.56°, 55.76° determined the n-BG [50]. The result of D value, using 2*θ*= 29.76°, was calculated at 46.6 nm.

**Fig. 3:**
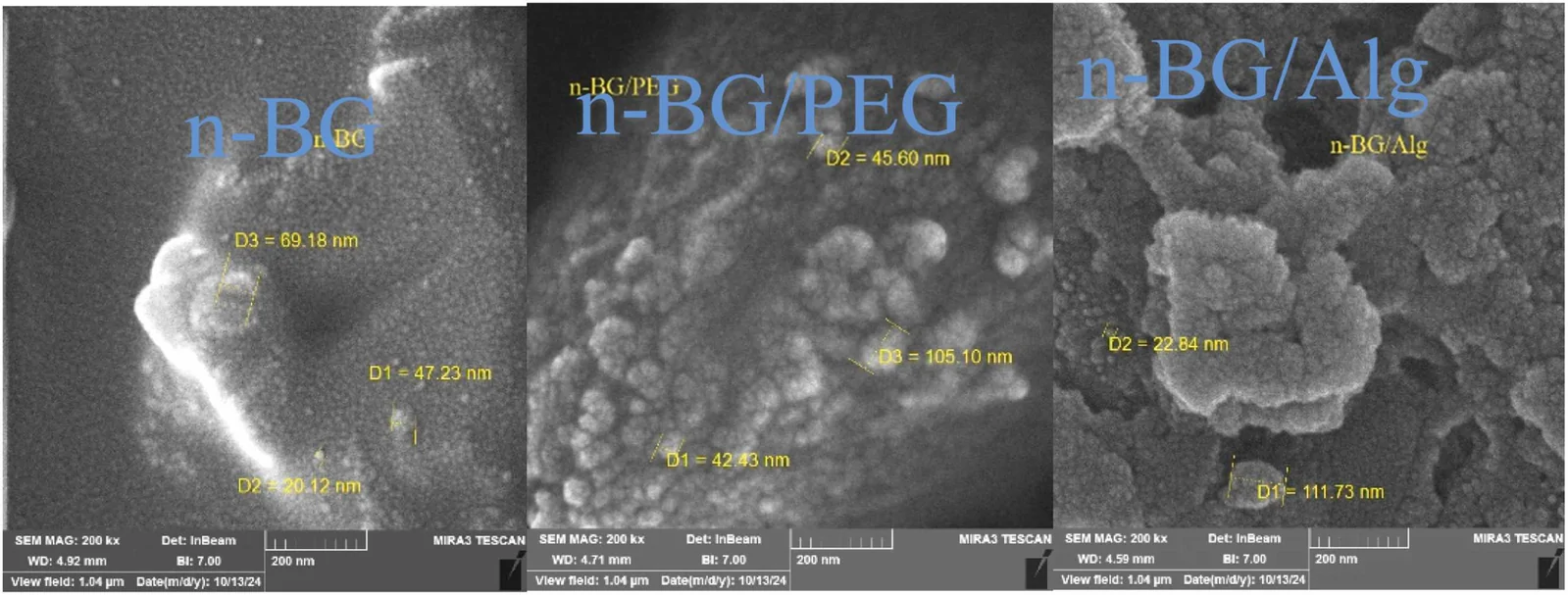
FESEM images of n-BG, n-BG/PEG and n-BG/Alg.

As can be seen, the XRD pattern of n-BG/Alg (Fig. 3b) shown peaks at 2*θ*= 29.4°, 31.96°, 39° and 48.16°. According the main peak at 2*θ*= 29.4, the size of n-BG/Alg was estimated 64 nm. Also, the XRD spectrum of alginate also indicates more crystalline nature than n-BG. XRD pattern of n-BG/PEG showed diffraction peaks at 2*θ*= 23.44°, 29.76°, 35.84°, 39.28°, 48.8°, 55.24°. The result of D value, using 2*θ*= 48.8°, was calculated at 102 nm.

### 3.3 FESEM & EDX

Field Emission Scanning Electronic Microscopy (FESEM) was used to investigate the morphology of the n-BG and nanocomposites surface. Fig. 3 indicates the pure n-BG has spherical and packed with smooth surface structure, and n-BG/Alg has more porosity with rough surface.

**Fig. 4:**
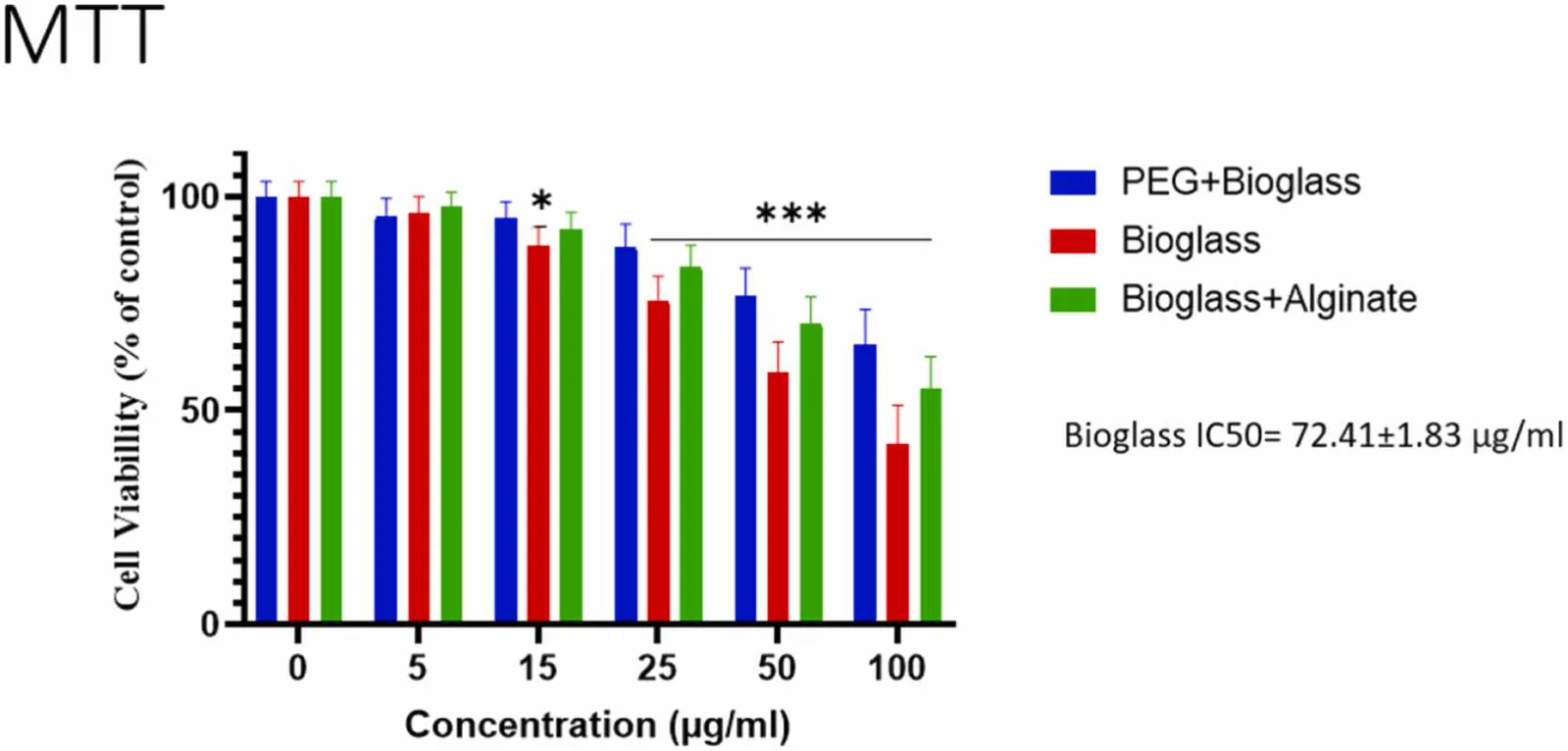
MTT assay of n-BG, n-BG/Alg, and n-BG/PEG, ^***^p < 0.001.

The diameter of n-BGs are estimated to be 22–65 nm and n-BG/Alg and n-BG/PEG are estimated to be 40–110 nm. As can be seen in the FESEM image Fig. 4b, many cluster-shaped aggregations with distributed pore structures are found in the nanocomposite scaffolds. FESEM clearly showed uniform and homogeneous matrices in the composite scaffolds, with porous morphologies and exhibited n-BG is dispersed within polymeric matrices.

### 3.4 Porosity and water uptake study

Porosity is important scaffold characterization for the distribution and transport of oxygen, nutrients and essential body fluids through scaffolds. In the present study, the porosity of BG nanocomposite scaffolds was determined by liquid displacement method employing Archimedes’ Principle. In the present study, the porosity of nano scaffolds was determined according liquid displacement method and the results showed n-BG/PEG has porosity approximately 81%, n-BG approximately 87% and n-BG/Alg approximately 74%. The results showed n-BG/PEG provide appropriate matrix for using as tissue scaffold.

The lowest porosity has been seen in n-BG/Alg, due to the intermolecular hydrogen bonding between O-H groups of alginate and BG. The porosity of the n-BG/PEG scaffold decreased almost up to 81%, due to the interaction between functional groups. This estimated porosity is appropriate for the cell proliferation, oxygen and nutrient migration in to the scaffolds. The water uptake, as defined to ability of water maintenance, is another important characterization of the scaffold in tissue engineering. According to this property, a scaffold might damage under excess water absorption [42], [51].

Water uptake of n-BG, n-BG/Alg, and n-BG/PEG was 29%, 25% and 27%, respectively. These results suggest that prepared scaffolds can absorb water up to 20% of their volume, and could swell to almost 20% of their size. These results could be demonstrated that the scaffolds are hydrophilic which facilitate the absorption of body fluid, and diffusion of nutrients [26].

### 3.5 MTT assay

The cell viability of these nanostructures were evaluated with 3-(4, 5-Dimethylthiazol−2-yl)−2, 5-diphenyl-tetrazoliumbromide (MTT) assay. As seen in Fig. 4, the cytotoxicity test of the nano scaffolds depict, in low concentrations, showed biocompatibility and low cytotoxicity. In high concentrations of scaffolds during 48 h, all scaffolds showed a significant difference (p˂0.001) compared to the positive control group. The MTT assay confirms that the scaffolds have low cytotoxicity to cells.

The results indicated that n-BG/PEG had biocompatibility. Also, all the above results suggest that the n-BG/PEG scaffold has good morphology and porosity for cell proliferation. It is noteworthy that many studies have clearly shown that n-BG/Alg and n-BG/PEG scaffolds are non-toxic and suitable for the attachment and growth of bone cells. Compared to other scaffolds, the scaffolds studied in this research have good biocompatibility and permeability.

Nano bioactive glass (n-BG) is widely used materials for tissue engineering due to their capability to form new tissue, strong bonding with the bone and controlled degradability. BG nanocomposite scaffolds are one of the most attractive biomaterials for tissue engineering. In this regard, our findings demonstrated that n-BG composite scaffolds were successfully synthesized by the sol-gel method as confirmed by FT-IR, XRD, FESEM and EDX analysis. As the characterization revealed, uniform nanostructures with smooth surfaces were formed. The results showed that the mean nanostructures diameter was increased by the addition of alginate and PEG to the n-BG compared to the uncoated n-BG.

By modifying the n-BG with polymers, the mean nanocomposites diameter was increased to 64, 102 nm. This is maybe the possible reason for the decrease in porosity of the scaffolds. Mukundan et al. (Mukundan et al., 2013) showed the ethanol washing of bioactive glass and conventional heat treatment calcination to stabilize bioactive glass. According to their findings, ethanol washing resulted in higher pore volume (total porosity 91%), diameter, and specific surface area [52]. Zhao et al. reported the sol-gel synthesis of 3D printing scaffold by nano bioactive glass modified with 10% polyvinyl alcohol (PVA) solution in a mass ratio of 1:1 for the dental implant [53]. However, our results showed that the incorporation of alginate and PEG to the n-BG matrix decreased the porosity compared to the pure n-BG. Also, the ultimate water uptake ability was decreased in the nanocomposite scaffolds. It is worth mentioning that the interaction between alginate and n-BG can be decreased the total porosity compared to others. These findings confirmed that n-BG/Alg can be a suitable scaffold for tissue engineering applications due to poor water absorption and probably, a slow degradation rate.

In our study, calcium nitrate was used precursor in the sol−gel preparation of bioactive glasses and nanocomposites, which needs to be calcinated at above 500 °C to remove toxic nitrate ions. Also, the MTT assay was used to evaluate the biocompatibility of scaffolds. For this assay, the Human Gingival Fibroblast (HGF) cell line, were used. Thus, it was of great importance to test the toxicity effect on HGF cells of scaffolds, which would be used as a bioactive composition to prepare tooth repair materials. Cell viability values were all normalized to the control sample at the sample cell culture intervals, thus values greater than 100% mean good biocompatibility. Bioactive glass with PEG had the highest bioactivity. As seen in Fig. 4, the n-BG did not have any negative effect on the growth of the cells in low concentrations, indicating they had no toxicity on HGF cells. Indeed, the group with n-BG/Alg even showed a better performance on the acceleration of the cell growth than the other groups within the incubation time, further confirming their good bioactivity. Thus, the addition of n-BG into the polymer enhanced the osteogenic differentiation.

Alginate’s carboxyl groups (COO^-^) interact with calcium ions (Ca^2+^) of n-BG and exchange with Na^+^ ions from external medium. This interaction cause to diffusion, drug release, crosslinking, and degradation properties of n-BG/Alg. This study was reported by Bider et al. for application of hydrogel composite of nBG-alginate by flame spray synthesis in room temperature [54]. However, Bargavi et al. reported a sol–gel assisted freeze-drying method for fabrication of Nbg/Alginate scaffold and the swelling, degradation and regeneration ability of scaffold were studied for tissue engineering [55].

Porosity and pore size as well as water uptake ability are very important properties for tissue engineering scaffolds, as they allow migration oxygen, nutrients and proliferation of the cells [56].

Moreover, high porosity with large pore size generally caused water absorption of the scaffolds. Therefore, the optimal values 80–85% require for porosity in order to facilitate cell migration and tissue ingrowth. Our finding was consistent with Srinivasan et al. and Naga et al., who suggested that alginate scaffold is biocompatible with human periodontal ligament fibroblast (hPDLF), osteosarcoma (MG−63) and HGF cells. This could be related to alginate interaction with n-BG [57], [58].

From these findings, we examined that n-BG/PEG showed the least pronounced toxicity. However, there is little research on n-BG/PEG. Zhou et al. prepared nano bioactive glass via sol-gel method and freeze-dried technique using polyethylene glycol (PEG) as templates and investigated the influences of molecular weight and content of polyethylene glycol on the morphology and the size NBG particles were investigated. Their results showed that the PEG molecular weight and PEG content have obvious effects on the morphology and size of the n-BG particles. They reported that the particle size of the n-BG particles decreased with the increase of PEG concentration [59].

There are some limitations of the present study. First, we used one concentration ratio of n-BG/Alg and n-BG/PEG at scaffold preparation without examining the effect of these ratio on particle size, morphology, and physicochemical properties. Additionally, we should be used different polymers and compare with each other. Future research involving different concentration ratios and type of polymer matrix should be performed to evaluate their effect, which are essential for accurate cytotoxicity and biocompatibility assessments.

## 4 Conclusion

Alginate/nBGC composite scaffolds were successfully fabricated using lyophilization technique and characterized. The scaffolds were found to have characteristic materialistic and biological properties essential to facilitate periodontal regeneration. The composite scaffolds had a pore size of about 100–300 m, controlled porosity and swelling ability, limited degradation and enhanced biomineralization, ideally controlled due to the presence of nBGC in the alginate scaffold. Incorporation of nBGC did not alter the viability of MG−63 and hPDLF cells and also helped to attain good protein adsorption, cell attachment and cell proliferation onto the scaffolds. The hPDLF cells also showed distinct osteoblast-like behavior with enhanced alkaline phosphatase activity. Our research suggested that alginate/nBGC composite scaffold can serve as an appropriate bioactive matrix for periodontal tissue regeneration.

## CRediT authorship contribution statement

**Mohammad Shokrzadeh:** Writing – original draft, Supervision, Resources, Project administration. **Masoumeh Eslamifar:** Writing – review & editing, Writing – original draft, Methodology, Formal analysis, Data curation. **Nazanin Farhadyar:** Writing – review & editing, Writing – original draft, Visualization, Methodology, Investigation. **Fereshteh Fathi:** Writing – review & editing, Writing – original draft, Methodology, Formal analysis, Data curation. **Gharekhan Elahe:** Writing – original draft, Software, Methodology, Data curation.

## Declaration of Competing Interest

The authors declare that they have no known competing financial interests or personal relationships that could have appeared to influence the work reported in this paper.

## Data Availability

Data will be made available on request.
